# Characterization of the Flavor Profiles of Fresh and Hot-Air-Dried Ginger (*Zingiber officinale* Roscoe) by Molecular Sensory Science

**DOI:** 10.3390/foods15132377

**Published:** 2026-07-03

**Authors:** Chenwei Li, Tianyu Dong, Panpan Wu, Jie Sun, Haitao Chen

**Affiliations:** 1 Beijing Key Laboratory of Flavor Chemistry, Beijing Technology and Business University, Beijing 100048, China; 14747951962@163.com (C.L.); dty19991030@163.com (T.D.); 18617240636@163.com (P.W.); chenht@th.btbu.edu.cn (H.C.); 2 Key Laboratory of Geriatric Nutrition and Health, Beijing Technology and Business University, Ministry of Education, Beijing 100048, China

**Keywords:** ginger (*Zingiber officinale* Roscoe), hot-air drying, GC-MS, molecular sensory science, key flavor compounds

## Abstract

Dried ginger slices are widely used for both culinary and medicinal applications. It is worth noting that a systematic model describing the dynamics of flavor profile changes during the drying of ginger has not been established. This study aimed to investigate the effects of hot-air drying on the volatile composition and odor-active compounds of ginger. Sensory evaluation revealed a shift in the aroma profile from pungent, floral, and herbal notes in fresh ginger toward predominantly roasted and woody notes in dried samples. In this study, molecular sensory science was employed. The results showed that 92 volatile compounds were tentatively identified, with terpenes showing a pronounced increase in relative proportion after drying. Nineteen compounds with flavor dilution (FD) ≥ 8 and odor activity value (OAV) ≥ 1 were confirmed as key aroma contributors, with zingiberene, linalool, geraniol, citronellal, 1,8-cineole, myrcene, and (*E*)-nerolidol exhibiting the highest FD values. These findings provide a quantitative basis for understanding aroma modulation during ginger drying and may offer practical guidance for the quality control of dried ginger products.

## 1. Introduction

Ginger (*Zingiber officinale* Roscoe) is a perennial herb of the *Zingiberaceae* family that has been cultivated for nearly 2000 years. It is widely used globally as both a culinary spice and a traditional medicinal plant, with documented applications in treating colds, stomach discomfort, diarrhea, and nausea [1,2,3]. At present, ginger is extensively cultivated in tropical and subtropical regions, including India, Nigeria, China, Nepal, Indonesia, Thailand, Peru, and Australia [4]. Among these, China is the largest producer and exporter, with exports valued at over US $800 million in 2024, accounting for the highest global share; major production areas are located in Shandong, Hebei, Liaoning, and Fujian provinces [5]. Shandong Province, in particular, is one of the key ginger-producing regions in China, where diverse cultivars are grown under favorable ecological conditions that contribute to their distinctive flavor and quality. The mature ginger used in this study was sourced from Weifang, Shandong Province, a major production area within this region.

In culinary applications, ginger is commonly used in both fresh and dried forms as an important seasoning ingredient, contributing to the characteristic flavor of various dishes. The flavor of ginger comes mainly from its non-volatile pungent compounds (e.g., gingerols and shogaols) and a complex mixture of volatile aroma compounds, with terpenes such as zingiberene and *β*-bisabolene being the dominant contributors to its aroma [6]. Owing to these properties, ginger is widely incorporated into a variety of food products, including sauces, beverages, and other processed foods. With the diversification of food processing and the increasing consumer demand for distinctive flavors, ginger has been utilized in different processed forms, which may result in variations in its flavor characteristics. Although some studies have compared the volatile profiles of fresh and dried ginger [7], the differences in flavor characteristics between fresh and dried ginger, as well as the associated changes in volatile compounds during the drying process, have not yet been systematically investigated.

Currently, research on ginger has mainly focused on its bioactive components, volatile flavor compounds, and processing technologies. In terms of flavor characterization, analytical techniques such as gas chromatography–mass spectrometry (GC-MS) have been widely applied, revealing that terpenes are the dominant volatile constituents in ginger, including zingiberene, *β*-sesquiphellandrene, and *α*-farnesene, with alcohols, aldehydes, and ketones also contributing to its aroma profile [8]. Processing, particularly drying, has been reported to significantly affect ginger flavor. Drying can alter the composition and content of volatile compounds, generally leading to a decrease in terpenes and changes in aroma characteristics [9]. Meanwhile, the transformation of gingerols into shogaols during thermal processing enhances the pungency of dried ginger [3]. In addition, different drying methods show varied effects on flavor retention, with techniques such as microwave- and silica gel-drying better preserving key aroma compounds compared with conventional methods [10]. Nevertheless, hot-air drying remains the predominant method of drying for agricultural crops [11]. It has the advantages of high efficiency and low cost. However, most existing studies have focused on the comparison of various drying methods, while systematic investigations that simultaneously characterize both volatile and non-volatile flavor differences between fresh and dried ginger using complementary analytical approaches remain limited. In summary, it is essential to employ molecular sensory science and technology to investigate the dynamics of flavor profiles and flavor compounds during the drying process of ginger at the molecular level.

In this study, volatile organic compounds (VOCs) in fresh ginger and dried ginger were extracted by solvent-assisted flavor evaporation (SAFE). Then, GC-MS and gas chromatography–olfactometry–mass spectrometry (GC-O-MS) combined with aroma extract dilution analysis (AEDA) were employed for the qualitative and quantitative analysis of volatile flavor compounds in fresh ginger and dried ginger. Subsequently, odor activity values (OAVs) were calculated to determine the contribution of each VOC to the overall aroma of the fresh ginger and dried ginger samples. This study provides a theoretical foundation for regulating and enhancing the flavor quality of hot-air-dried ginger products.

## 2. Materials and Methods

### 2.1. Materials

Mature ginger samples (from Weifang City, Shandong Province, China) were purchased from Meituan Xiaoxiang Supermarket, an online grocery platform in Beijing, China.

### 2.2. Chemicals

The C5–C28 n-alkane mixture (≥99.9%, GC grade) was purchased from Sigma-Aldrich (Shanghai, China). Dichloromethane (analytical grade, ≥95%) and anhydrous sodium sulfate (≥95%) were obtained from Sinopharm (Beijing, China). Dichloromethane was redistilled before use. Individual aroma compound standards were supplied by Beijing InnoChem Science & Technology Co., Ltd. (Beijing, China) and Beijing Mreda Technology Co., Ltd. (Beijing, China). All other chemicals were of analytical or chromatographic grade.

### 2.3. Ginger Sample Preparation

Fresh ginger was thoroughly washed to remove adhering sand, and then blotted with absorbent paper to remove excess surface water. The cleaned ginger was sliced into pieces of uniform thickness (2 ± 0.2 mm) using a multi-function vegetable cutter (Supor, Hangzhou city, Zhejiang Province, China). A separate representative sample of the fresh slices was used to determine the initial moisture content by drying in a hot-air oven at 65 °C to constant weight; the value was 95.22 ± 0.01% on a wet basis.

For the drying experiment, the ginger slices were divided into 15 portions of 40 ± 0.5 g. Three portions were immediately set aside as the reference sample (sample 1), representing the fresh ginger prior to any drying. The 12 drying portions corresponded to four drying time points (0.5, 1.0, 1.5, and 2.0 h), with three independent replicate portions per time point. At each designated time, the corresponding samples were taken out of the oven for subsequent analysis. Based on a comprehensive review of the literature, 65 °C was identified as the optimal drying temperature for ginger [12]. The total drying time was limited to 2 h, which corresponded to the time required for the moisture content to decrease to approximately 10%; this endpoint is widely adopted in related studies [13]. Consequently, five sample sets were defined: fresh (0 h) and dried for 0.5, 1.0, 1.5, and 2.0 h, designated as samples 1–5, respectively. These samples were arranged in a single layer on separate trays without overlap, and were dried simultaneously in a hot-air oven (Forced-air drying oven, Beijing Tianlin Hengtai Technology Co., Ltd., Beijing, China) at 65 ± 1 °C. Detailed drying parameters are provided in Table S1.

### 2.4. Assessment of Overall Aroma Profile of Ginger

Quantitative descriptive analysis (QDA) was employed to characterize the aroma profiles of ginger samples subjected to the five drying intervals described in Section 2.3. Twelve panelists (eight females, four males, aged 20–30) were recruited from the Beijing Key Laboratory of Flavor Chemistry at Beijing Technology and Business University. All participants provided written informed consent, were pre-screened for normal olfactory function, and reported no history of respiratory or olfactory diseases. Panel training was conducted over one week. During the sessions, panelists were first presented with the full set of ginger samples and asked to generate odor descriptors individually. Through group discussion, the most frequently cited terms were consolidated, and by consensus, eight attributes were selected to define the aroma space: sweet, pungent, floral, fruity, woody, herbal, roasted, and grassy. The eight attributes were defined as the following flavor references: vanillin (sweet), linalool (pungent), geraniol (floral), limonene (fruity), cedrol methyl ether (woody), camphor (herbal), 2-methylpyrazine (roasted), and leaf alcohol (grassy). The concentrations of the aroma references were 100 times their respective odor thresholds in water.

Formal evaluations took place in a dedicated sensory laboratory maintained at 25 °C. Samples (5.0 g) were placed in 30 mL plastic bottles, capped for 10 min to allow for headspace equilibration, and then presented in a randomized order. For each sample, panelists sniffed the headspace and rated the intensity of the eight pre-defined attributes on a 10-point category scale, where 0 = not detectable, 5 = moderate, and 9 = extremely strong.

This sensory evaluation experiment was approved under Certificate No. 62 of 2026 issued by the Scientific Research Ethics Committee of Beijing Technology and Business University.

### 2.5. SAFE/GC-MS Analysis

Volatile compounds were isolated using SAFE. SAFE can accurately extract complete volatile flavor compounds from complex food matrices under mild conditions of low temperature and high vacuum. Each ginger sample was accurately weighed, then frozen in liquid nitrogen and ground to a fine powder (exact masses recorded in Table S1). The frozen powder was transferred into a 500 mL separatory funnel, followed by the addition of 200 mL of redistilled dichloromethane and 10 μL of 4-nonanol (41.5 mg/mL final concentration in the extract) as an internal standard. The mixture was shaken on a GGC-C separating funnel vertical shaker (Beijing Guohuan High tech Automation Technology Research Institute, Beijing, China) at 300 rpm for 30 min, with venting performed every 10 min to release internal pressure, and then filtered under vacuum through filter paper. The filtrate was subjected to SAFE distillation under high vacuum (<10^−4^ mbar) with a circulating water bath at 50 °C. The resulting distillate was dried by adding anhydrous sodium sulfate portionwise until no visible water droplets remained on the liquid surface, and then left to stand for approximately 12 h. After filtration, the extract was concentrated to approximately 2 mL using a 50 cm × 1 cm Vigreux column, and then further concentrated to about 1 mL under a gentle stream of nitrogen. The final extract was stored in a refrigerator at −40 °C for backup.

GC-MS analysis was performed following a previously reported method with minor modifications [14], using a Trace 1310 gas chromatograph coupled to a mass spectrometer (Thermo Fisher Scientific, Waltham, MA, USA). Volatile compounds were separated on a DB-Wax capillary column (30 m × 0.25 mm × 0.25 μm, Thermo Fisher Scientific). Helium was used as the carrier gas at a constant flow rate of 1.0 mL/min. The injection port temperature was set at 250 °C, and a sample volume of 1.0 μL was injected with a split ratio of 20:1. The GC oven temperature program was as follows: an initial temperature of 50 °C held for 5 min; raised to 100 °C at 3 °C/min, then to 160 °C at 2 °C/min, and finally to 220 °C at 5 °C/min; and held for 3 min. The mass spectrometer was operated in electron ionization (EI) mode at 70 eV. The ion source and transfer line temperatures were both maintained at 230 °C. After a solvent delay of 5 min, mass spectra were acquired in full scan mode over the range *m*/*z* 30–350.

### 2.6. GC-O-MS Analysis

Odor-active compounds were characterized by GC-O-MS using a GC-MS system (7890B Gas Chromatography System equipped with 5977B GC/MSD (Agilent Technologies Inc., Santa Clara, CA, USA)). The same chromatographic column and oven temperature program as described for GC–MS analysis (Section 2.3) were applied. After chromatographic separation, the column effluent was split 1:1 (by volume) between the mass spectrometer and the olfactory detection port. Prior to the GC-O-MS analysis, the three experts (selected from the panel in Section 2.4) received specific training on ginger sensory descriptors and the odor attributes of its key odor-active compounds. Leveraging their extensive experience in olfactory evaluation, they recorded the time, intensity, and characteristics of each odor during the analysis. A compound was considered odor-active when it was detected by at least two of the three panelists.

### 2.7. Aroma Extract Dilution Analysis (AEDA)

To identify the most potent odor-active compounds, the concentrated SAFE extracts of two representative samples—fresh ginger (sample 1) and ginger dried for 2.0 h (sample 5), representing the initial and the most intensely dried states—were subjected to AEDA. Three trained panelists from the sensory panel (Section 2.2) participated. Each extract was progressively diluted with dichloromethane at a stepwise ratio of 1:1, yielding dilution steps of 2^*n*^ (*n* = 0, 1, 2, 3, …). Each dilution was analyzed by GC-O under the same conditions as described above. Panelists recorded the odor characteristics of each perceived odorant, along with its corresponding retention time. The dilution process continued for each odor until it was no longer perceived by at least two of the three panelists. The flavor dilution (FD) factor was defined as the highest dilution at which the odor was still detected by a minimum of two panelists. Compounds with the highest FD factors were considered the most significant contributors to the overall aroma.

### 2.8. Qualitative and Quantitative Analyses of Volatile Compounds

Volatile compounds were identified by matching their mass spectra with the NIST17 database and by comparing their calculated retention indexes (RIs) with published values. For RI calculation, a homologous series of C5–C28 n-alkanes were analyzed under the same chromatographic conditions as the samples. Finally, the compounds were validated against authentic aroma standards under identical chromatographic conditions.

All volatile compounds tentatively identified by matching with the NIST17 database were first subjected to semi-quantitative analysis using 4-nonanol (41.5 mg/mL, final concentration in the extract) as an internal standard. Based on the results of AEDA, volatile aroma compounds with FD ≥ 8 were selected for further quantification using the internal standard curve method. The concentration ranges for the standard curve were informed by the semi-quantitative results: for each target compound, the approximate concentration estimated from the semi-quantitative data was used as a reference point, and a series of seven standard solutions were prepared by gradient dilution with dichloromethane to bracket this value. Each calibration solution contained the internal standard at the same final concentration as the samples (41.5 mg/mL). These solutions were analyzed by GC-MS under the same chromatographic conditions described in Section 2.3, operating in selected ion monitoring (SIM) mode. Standard curves were constructed by plotting the peak area ratio (analyte/internal standard) versus the concentration ratio (analyte/internal standard). All curves exhibited good linearity with correlation coefficients (R^2^) ≥ 0.99. The residual plots are shown in Figure S1. Each quantification was performed in triplicate, and the mean values are reported.

### 2.9. Determination of OAVs

The OAV was calculated for each of the aroma compounds quantified by the internal standard calibration method (i.e., those with FD ≥ 8 in AEDA) to evaluate its individual contribution to the overall aroma profile. The OAV was defined as the ratio of the compound’s concentration, determined from the calibration curve, to its odor detection threshold in water. Compounds with OAV ≥ 1 were considered to contribute directly to the characteristic aroma of the sample. Odor threshold values were obtained from the published literature [15].

### 2.10. Recombination Experiments

Multiple extractions of fresh ginger were carried out with organic solvents, including dichloromethane and n-pentane, to remove endogenous aromatic components and prepare an odorless ginger matrix. Subsequently, the aroma compounds with OAV ≥ 1 were added to the odorless matrix in amber glass vials at their quantified concentrations: 30 compounds for the fresh ginger reconstitution and 27 for the dried ginger reconstitution. The sensory evaluation panel was composed of 10 assessors described in Section 2.2. All panelists further evaluated the olfactory similarity between the reconstituted sample and the original ginger sample via sensory analysis.

### 2.11. Statistical Analyses

Results were expressed as mean ± standard deviation (SD) from at least three independent replicates. One-way analysis of variance (ANOVA), followed by Duncan’s multiple range test, was conducted using IBM SPSS Statistics 27.0.1 (IBM Corp., Armonk, NY, USA) to evaluate significant differences among samples at a threshold of *p* < 0.05. Correlation analysis and two-dimensional plotting were performed using Origin 2024b (OriginLab Corp., Northampton, MA, USA). Multivariate statistical analysis, including principal component analysis (PCA) and orthogonal partial least squares-discriminant analysis (OPLS-DA), was carried out in SIMCA 14.1 (Umetrics, Umea, Sweden). Hierarchical clustering heatmaps were generated using TBtools v2.0.

## 3. Results and Discussion

### 3.1. Differences in the Overall Aroma Profiles of the Ginger Drying Process

**Figure 1:**
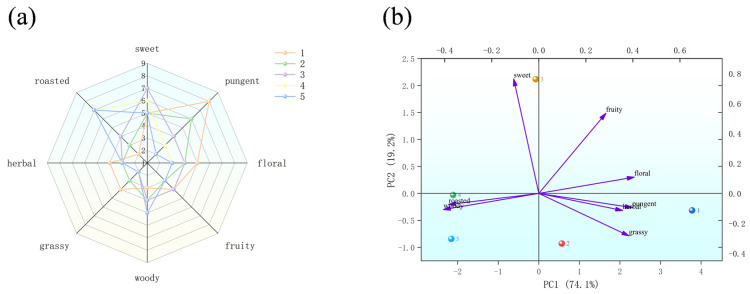
Aroma characterization of ginger. (**a**) The flavor profile description; (**b**) PCA plot of ginger aroma descriptions. Fresh (0 h) and dried for 0.5, 1.0, 1.5, and 2.0 h are designated as samples 1–5, respectively.

The aroma profiles of ginger samples at different drying stages were evaluated using QDA, and the results are presented in Figure 1a. As shown in the radar chart, fresh ginger (Sample 1) was dominated by strong pungency, and grassy and herbal notes, along with moderate floral attributes, which constitute the typical sensory properties of fresh ginger for daily culinary seasoning. With increasing drying time, the intensities of pungent, floral, grassy, and herbal aromas gradually declined, indicating a progressive loss of the fresh aroma character. In contrast, the roasted note exhibited an opposite trend, becoming increasingly prominent as drying proceeded, particularly in samples subjected to longer drying durations (1.5–2.0 h). Notably, the aroma intensities of pungent and roasted attributes presented a striking inverse correlation with the extension of drying time, suggesting that these two aroma attributes were highly characteristic of the drying-driven sensory shift. Among all samples, the ginger dried for 1.0 h (Sample 3) possessed the maximum sweetness intensity. This observation suggests that appropriate mild drying conditions could facilitate the generation and release of volatile compounds responsible for the sweet note. Meanwhile, woody and fruity notes remained relatively low across all samples, with only minor and insignificant fluctuations during the drying process. Overall, the results demonstrate a clear shift in aroma profile from fresh and pungent attributes to more thermally derived roasted characteristics as drying time increased.

PCA was performed to explore the aroma differences among the five ginger samples with varying drying times. As shown in Figure 1b, fresh ginger (Sample 1) displayed a distinct distribution pattern, characterized by strong grassy, pungent, and herbal aromas, separating it clearly from the dried samples along the PC1 axis. In contrast, the dried samples showed a gradual shift in aroma profiles with increasing drying time, as evidenced by their movement along the PCA axes. Sample 2, which had the shortest drying time, exhibited a balance between grassy and woody aromas, suggesting a moderate transformation of its fresh attributes. Sample 3 was distinguished by its high score on PC2, where the sweet attribute exhibited the strongest loading, pointing to a close association with sweet aroma notes under this specific drying condition. Samples 4 and 5 were both positioned at the negative extreme of PC1, closely associated with roasted and woody attributes, indicating the most pronounced thermal-processed aromas among all samples. These PCA findings align well with the QDA results, confirming that the overall aroma variation during drying is primarily driven by the shift from fresh and pungent notes to thermally derived roasted characteristics, while a transient enhancement of sweetness occurs at an intermediate drying stage (1.0 h).

### 3.2. Profiling and Relative Composition of Volatile Compounds in Ginger During Drying

**Figure 2:**
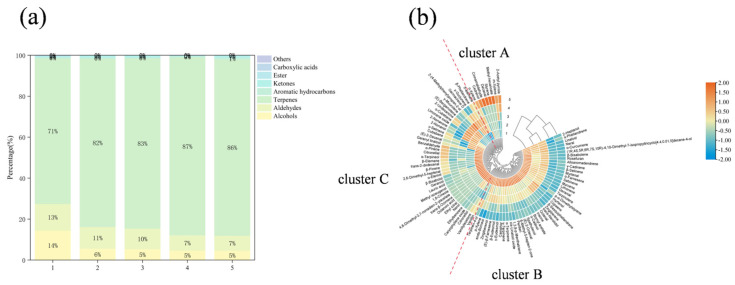
Analysis of GC–MS data. (**a**) Relative content of compounds in ginger; (**b**) heatmap analysis of aroma compounds in ginger. The numbers correspond to the compound Nos. in Table 1.

To investigate the aroma changes of ginger during drying, SAFE combined with GC-MS analysis was used to identify the composition and relative content of aroma compounds from ginger with different drying times (Table 1). A total of 92 volatiles were identified by the qualitative method on a polar (DB-WAX) capillary column combined with standards. During the drying process of ginger, the percentage of terpenes increased from 71% to 86%, while aldehydes and alcohols decreased (Figure 2a).

To further elucidate the effect of drying time on ginger aroma compounds, a clustered heatmap combined with hierarchical cluster analysis (HCA) was used to visualize the relative contents of 92 aroma substances in Table 1, as shown in Figure 2b. The data were Z-score normalized, and the color change from blue to orange reflects the change in volatile compound content from low (−2) to high (2). The heatmap revealed three abundance trajectories across the five drying stages. Cluster A compounds increased progressively, reaching their highest levels at the later stages; this accumulation likely resulted from a combination of water loss-induced concentration and thermal generation via Maillard-type pathways [16,17]. In contrast, Cluster B compounds declined markedly over drying time, consistent with losses of highly volatile or thermolabile constituents [18]. Cluster C exhibited relatively stable levels throughout the process, indicating higher thermal stability. These contrasting patterns demonstrate that drying significantly and selectively restructures the aroma profile of ginger, with some odor-active compounds being enriched while others are substantially depleted, resulting in divergent abundance profiles.

The formation of volatile flavor compounds in ginger involves two interconnected pathways. The first is a biosynthetic route that operates during rhizome growth and maturation, where lipids, amino acids, and specific secondary metabolite precursors are enzymatically converted into volatile metabolites [19,20], either through direct transformation (e.g., hydrolysis, transferase reactions) or through enzyme-mediated oxidative cleavage [21]. The second is a non-enzymatic, chemical pathway that predominates during postharvest processing: exposure to thermal treatments or fermentation triggers reactions such as thermal degradation, Maillard reactions, and oxidation [3], which further transform pre-existing precursors and newly generated intermediates into the volatile profile characteristic of processed ginger.

Terpenes constitute the predominant volatile fraction of ginger, accounting for over 60% of the total volatile profile, and their pronounced volatility underpins the characteristic aroma of ginger. Key terpenes such as zingiberene, *β*-bisabolene, *α*-farnesene, and terpinolene jointly impart the spicy, woody, citrus, and subtle floral notes typical of ginger [1]. The biosynthesis of these compounds proceeds via the cytosolic MVA and plastidial MEP pathways, which converge at IPP and DMAPP; downstream, a family of 25 mono- and 18 sesquiterpene synthases catalyzes the assembly of the diverse terpenoid scaffolds detected in ginger essential oil [19,22].

During drying, the decline in alcohols and aldehydes passively inflated the terpenes share, even though most terpenes declined in absolute concentration [23]. Two complementary mechanisms likely account for this relative enrichment. First, fresh ginger contains substantial pools of glycosidically bound aroma precursors—including *β*-glucopyranosides of geraniol, nerol, linalool, and citronellol—that are enzymatically cleaved by endogenous glycosidases upon tissue disruption, releasing the corresponding volatile aglycones [24,25]. Second, thermal dehydration disrupts the structural integrity of oil cells and secretory cavities, facilitating the liberation of pre-formed terpenes that are otherwise compartmentalized within intact tissue [23]. The relative contribution of each mechanism probably depends on drying temperature, duration, and the degree of physical disruption.

Aldehydes represent the second most abundant volatile class in ginger. Their content declines during drying, partly due to evaporative loss under prolonged heating [9]. The predominant citral isomers, neral and geranial, are especially susceptible to thermal oxidation, which generates alcohols, epoxides, and smaller carbonyl fragments through allylic oxidation and double-bond cleavage [26,27]. Elevated temperature and reduced water activity in the later stages of drying may also promote Maillard-type reactions between residual aldehydes and amino acids or reducing sugars, further depleting the measurable aldehyde pool [28].

Alcohol losses during drying are similarly multifaceted. In addition to volatilization, monoterpene alcohols undergo partial conversion to their corresponding acetates [29]. Concurrently, the same glycosidase activity that liberates terpene aglycones also releases odor-active alcohols such as geraniol, nerol, linalool, and *α*-terpineol from their bound precursors; however, the contribution of this enzymatic pathway may diminish during prolonged drying as the responsible glycosidases undergo progressive thermal inactivation [24,25]. Among the alcohols detected in ginger, linalool, geraniol, nerol, citronellol, *α*-terpineol, trans-nerolidol, and 2-heptanol have been confirmed as key aroma contributors [25,30,31], collectively providing the floral, citrus, woody, and herbal notes that define the aromatic complexity of dried ginger products.

Collectively, these transformations—enrichment of terpenes, progressive loss of aldehydes through volatilization and oxidation, and the dual modulation of alcohols by acetate conversion and glycosidase-limited release—reshape the overall aroma profile of ginger during drying.

### 3.3. Differences in Aroma Compounds of Ginger at Different Drying Stages

**Figure 3:**
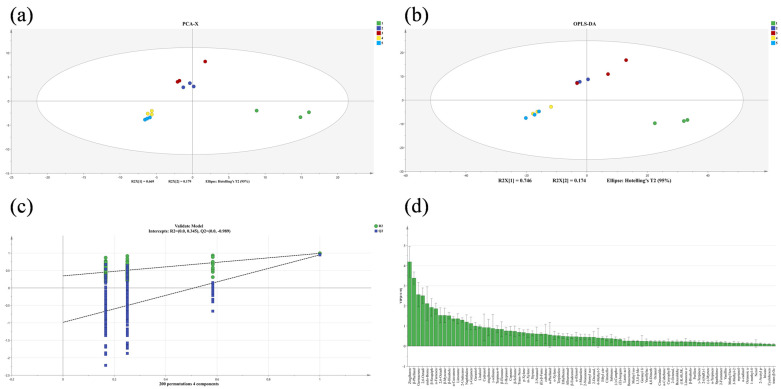
Detected volatile compounds in ginger. The contents of 92 volatile compounds in Table 1 were analyzed. (**a**) PCA score plots; (**b**) OPLS-DA score plots; (**c**) OPLS-DA model permutation test plots mode; (**d**) VIP plots.

We performed PCA on the aroma compound profiles to examine differences among fresh and variously dehydrated ginger samples [32]. In the PCA, the quantified aroma compounds were used as input variables, with sample groups labeled according to drying intensity; the resulting score plot is shown in Figure 3a. The model yielded an R^2^X of 0.943. The PCA-X score plot showed the separation and clustering of aroma compositions across the five groups. Group 1 was distinctly separated from the others along PC1, indicating its markedly different aroma profile. Groups 2 and 3 formed a compact cluster in the positive semi-axis of PC2 and near the center of PC1, whereas Groups 4 and 5 clustered closely in the negative semi-axis of both PC1 and PC2, demonstrating high similarity within each pair. The first two principal components captured 84.8% of the total variance (PC1: 66.9%, PC2: 17.9%). The tight clustering of biological replicates within each group reflected high reproducibility. Moreover, all samples fell within the 95% Hotelling’s T^2^ ellipse, indicating the absence of strong outliers.

To further characterize the volatile profile differences between fresh and dried ginger samples, an OPLS-DA was performed on the 92 volatile compounds listed in Table 1. OPLS-DA separates the predictive (inter-group) variation from orthogonal (intra-group) variation, concentrating the class-discriminating information into the first predictive component to improve model interpretability [33]. Unlike PCA, OPLS-DA provides the advantage of explicitly separating out systematic variation unrelated to the treatment, thereby enabling a more reliable identification of markers genuinely responsible for inter-group differences. This supervised approach has been effectively employed in volatile fingerprinting studies for sample discrimination and key marker screening via VIP values [34]. However, the application of OPLS-DA to high-dimensional datasets with limited sample size carries an inherent risk of overfitting. To address this, we performed rigorous internal cross-validation and permutation testing (*n* = 200), and restricted variable selection to compounds with VIP > 1.0 to enhance model robustness. The model yielded an R^2^X of 0.986, an R^2^Y of 0.922, and a Q^2^ of 0.756 (Figure 3b). The high R^2^Y and Q^2^ values indicated a satisfactory goodness-of-fit and robust predictive ability. The permutation test (200 permutations) yielded a Q^2^ intercept of −0.989, with all permuted Q^2^ values lower than the original one (Figure 3c), confirming that the model was not overfitted [35]. Compounds with a variable importance in projection (VIP) value greater than 1 and a univariate *p*-value of less than 0.05 were considered potential discriminatory markers [36]. Based on these criteria, 17 differential compounds were screened, with VIP values ranging from 1.0 to 4.2 (Figure 3d). Among them, zingiberene (VIP = 4.2), *β*-phellandrene (VIP = 3.4), germacrene D (VIP = 2.6), and geranial (VIP = 2.5) contributed most significantly to the classification. Importantly, these discriminatory markers only reflect differences in detectable abundance, and do not provide a direct measure of their actual contribution to the overall aroma. Therefore, a definitive identification of the key odor-active compounds in ginger would benefit from integrating molecular sensory science approaches, such as AEDA [37].

### 3.4. Aroma Extract Dilution Analysis (AEDA)

To further clarify the effect of drying on the odor-active components of ginger, both fresh ginger (sample 1) and samples subjected to two hours of drying (sample 5) were investigated. Volatile compounds were isolated by SAFE and subsequently analyzed by GC-O-MS. As summarized in Table 2, a total of 55 odor-active compounds were tentatively identified by comparing their mass spectra, RIs, and odor characteristics with reference standards and data from the NIST 17 library. The contribution of each odor-active compound to the overall ginger flavor was assessed by AEDA, expressed as the FD factor; a higher FD value signifies a more pronounced contribution [38]. Ten odor-active compounds were found exclusively in fresh ginger, whereas 14 were unique to the sample subjected to two hours of drying. Geraniol, zingiberene, (*E*)-2-dodecenal, and citronellal exhibited the highest FD values and served as the primary odor-active compounds. Thus, they might play a significant role in the formation of the characteristic ginger flavor. This result is consistent with those of recent studies [39]. Several other compounds also exhibited relatively high FD factors, including linalool (512; citrus, floral, sweet), *α*-cubebene (512; herbal, waxy), (*E*)-nerolidol (256; floral, citrus, woody, waxy), *α*-terpineol (256; woody, floral), *β*-myrcene (256; peppery, spicy, balsam), copaene (256; woody, spicy, honey), octanal (256; waxy, citrus, herbal, fatty), neral (256; sweet, lemon), (*E*,*Z*)-2,6-Dodecadienal (256; citrus), 2,6-dimethyl-5-heptenal (256; sweet, fruity, minty), and (*Z*)-linalool oxide (256; earthy, floral, woody). These volatiles are widely recognized as the principal contributors to the unique flavor profile of ginger [6].

It is noteworthy that a distinct roasted and burnt note was perceived in the two-hour-dried ginger samples. However, aside from 2-acetylpyrrole (FD 16, musty, nut, cherry, walnut), no compounds imparting such sensory attributes were detected during AEDA. This discrepancy can be more plausibly attributed to the limitations inherent in the extraction and analytical procedures. Odorants associated with roasted and burnt aromas are often highly volatile and thermally labile, and they may have been lost during high-vacuum SAFE distillation or degraded at the heated GC injection port [40]. Additionally, these trace-level odorants could be masked by co-eluting major aroma compounds, thereby escaping GC-O detection even though they contribute to the overall sensory impression of the sample.

Among the 23 compounds that exhibited decreased FD factors after drying, nine showed a significant decline: 2-nonanol, (*E*)-nerolidol, *α*-terpineol, myrcene, copaene, (*E*)-2-dodecenal, (*E*,*Z*)-2,6-Dodecadienal, 2,6-dimethyl-5-heptenal, and (*Z*)-linalool oxide. The FD factors of these nine compounds exhibited a 4- to 64-fold reduction; for instance, myrcene decreased from 256 to 64 and (*E*)-2-dodecenal from 1024 to 128. This selective loss underscores the high sensitivity of terpenes and unsaturated aldehydes to thermal processing. The marked reduction in terpenes is consistent with their known thermal lability and oxidative susceptibility. Under hot-air drying, *α*-terpineol, (*E*)-nerolidol, and (*Z*)-linalool oxide can undergo acid-catalyzed dehydration and rearrangement to form other terpenes, as previously documented in dried herbs [41,42]. Myrcene, with its conjugated diene structure, is particularly prone to auto-oxidation and evaporative loss, which explains its sharp decline even at moderate temperatures [10,42]. For the unsaturated aldehydes (*E*)-2-dodecenal and (*E*,*Z*)-2,6-Dodecadienal, a specific enzymatic pathway is likely involved: dehydrogenases, which remain active during the early stage of drying, can reduce these aldehydes to their corresponding alcohols, leading to a rapid loss of aldehyde-type aroma [5]. Collectively, these findings demonstrate that the loss of key odor-active compounds during ginger drying is governed by a complex interplay of thermal degradation, enzymatic conversion, and physical volatilization. The unexpected magnitude of aldehyde loss via enzymatic reduction, in particular, highlights the need to consider early-stage biochemical activity when designing drying protocols aimed at preserving ginger’s characteristic aroma.

The AEDA data revealed that most aldehydes exhibited higher FD factors in the two-hour-dried ginger than in the fresh sample, indicating that drying enhanced the sensory contribution of these odorants. This increase can be explained by several mutually reinforcing mechanisms. First, drying disrupts cellular integrity and promotes enzymatic lipid oxidation via the lipoxygenase (LOX) pathway, generating potent medium-chain aldehydes such as octanal, decanal, and (*E*)-2-decenal, which possess extremely low odor thresholds and contribute fatty, green, and citrus notes [43]. Second, thermal and dehydration stresses may facilitate the release of aldehydes from glycosidically bound precursors. The presence of glycosidically bound and phenylpropanoid aroma precursors in ginger has been documented [24,25]. In model-system studies, acid- or heat-catalyzed hydrolysis of structurally analogous glycosides—such as citronellyl, myrtenyl, and neryl glucosides—has been shown to directly generate the corresponding aldehydes, including citronellal, myrtenal, and neral [44]. Cinnamaldehyde, a phenylpropanoid aldehyde, may similarly arise from hydrolysis of its glycosidic precursor. Third, the substantial loss of high-abundance but less odor-active volatiles during drying diminishes olfactory competition during GC-O. Drying has been shown to drastically alter the volatile profile of ginger, with both losses of native compounds and the appearance of new volatiles [9], and substantial volatile depletion was consistently observed irrespective of the drying method employed [3]. Under these conditions, surviving low-threshold aldehydes become more distinctly perceivable to the GC-O assessor, thus receiving elevated FD scores even without an absolute increase in concentration. Collectively, the elevated FD values of octanal, decanal, (*E*)-2-decenal, myrtenal, citronellal, neral, and cinnamaldehyde point to a drying-induced reshaping of the aroma profile, reinforcing the green, citrusy, and spicy character of dried ginger.

### 3.5. Quantification and OAV Calculation

The 31 odor-active compounds with FD factors ≥ 8 were quantified by the internal standard curve method, and the results are summarized in Table 3. Their concentrations spanned a wide range, from 0.03 μg/g (cinnamaldehyde) to 1789.87 μg/g (zingiberene). In addition to zingiberene, several other compounds were found at high levels, notably 1,8-cineole (1006.89 μg/g), *α*-farnesene (861.27 μg/g), and neral (241.47 μg/g). However, the mere abundance of a volatile does not fully dictate its sensory impact.

The OAV, calculated as the ratio of the concentration of a compound to its odor detection threshold, provides a more reliable estimate of its contribution to the overall aroma profile [45]. Compounds with an OAV > 1 are generally regarded as key aroma compounds. Given that odor thresholds were available for only a subset of the 31 quantified odorants, OAVs were calculated exclusively for those compounds; among these, 21 showed an OAV > 1 in fresh ginger compared to only 16 in the sample dried for two hours. This decrease in the number of odorants with OAV > 1 was consistent with the observed reduction in the concentrations of all odorants after drying, although some key odorants could not be evaluated due to missing thresholds. In contrast, several compounds, including *γ*-terpinene, camphor, bornyl acetate, 2-acetylpyrrole, and cinnamaldehyde, displayed OAVs below 1, indicating that their direct contributions to the ginger flavor were negligible—a consequence of either high odor thresholds, low concentrations, or a combination of both. Convergent evidence from the OAV and AEDA analyses reinforces the identification of the most critical aroma contributors in ginger. With the exception of 1,8-cineole, all of the high-OAV compounds (defined as those with OAV > 100) also exhibited high FD factors, including linalool (OAV 4017, FD 512), geraniol (OAV 2568, FD 1024), neral (OAV 2414.7, FD 64), octanal (OAV 314, FD 64), (*E*)-2-dodecenal (OAV 105, FD 1024), myrcene (OAV 661, FD 256), 2-heptanol (OAV 250, FD 64), and (*E*)-nerolidol (OAV 181, FD 256). These compounds are therefore considered critical to the characteristic ginger aroma, imparting citrus, herbal, spicy, and sweet notes. This cross-validation by two independent sensory-directed methodologies reduces the risk of misjudgment that might arise from relying solely on OAV or AEDA.

The case of 1,8-cineole, however, merits separate discussion. Despite its high OAV (1006.89), its performance in AEDA was less prominent than would be predicted from its abundance. A plausible explanation lies in the well-documented limitations of conventional one-dimensional GC-O. The analysis of complex natural product matrices by GC-O is frequently compromised by co-elutions, which can lead to the masking of odor-active trace-level compounds by major interferences or the agglomeration of multiple olfactive impressions into an ambiguous odor cluster [46]. Given the inherent chemical complexity of ginger volatile extracts, it is conceivable that 1,8-cineole co-eluted with one or more abundant terpenes during GC separation, thereby suppressing its perceived odor intensity at the sniffing port. Notably, 1,8-cineole has been identified as among the most potent odorants by AEDA (FD 2187) in certain ginger studies employing complementary headspace-based olfactometry [6], and as a key odorant critical to the aroma profile in other botanical matrices through omission experiments [47]. These reports support the intrinsic odor potency of 1,8-cineole and indirectly point to matrix- or method-dependent factors, rather than a genuine lack of sensory relevance, as the reason for the discrepancy observed here.

Taken together, the combined OAV and AEDA evidence identifies zingiberene, linalool, geraniol, neral, citronellal, *α*-cubebene, *α*-terpineol, copaene, (*E*,*Z*)-2,6-Dodecadienal, 2,6-dimethyl-5-heptenal, (*Z*)-linalool oxide, 1,8-cineole, octanal, (*E*)-2-dodecenal, myrcene, and (*E*)-nerolidol as the principal odor-active volatiles responsible for the characteristic flavor of ginger. The agreement between the two methods for the majority of these compounds provides a robust foundation for future flavor reconstitution and omission studies.

### 3.6. Aroma-Recombination Experiments

**Figure 4:**
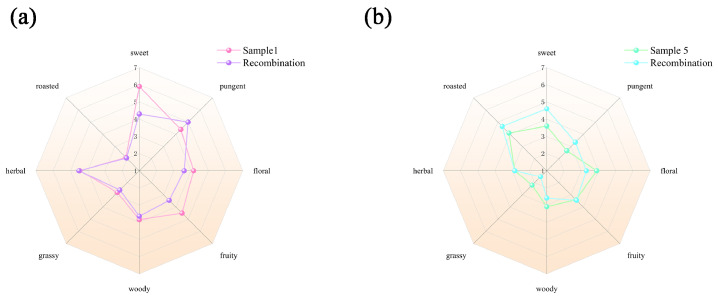
(**a**) Aroma characterization radargrams for sample 1; (**b**) aroma characterization radargrams for sample 5.

Chromatographic separation resolves individual odorants but cannot capture the perceptual interactions that arise in a complex mixture [48]. Aroma-recombination experiments are therefore essential to verify the key odor-active compounds in ginger. In this study, two independent recombination models were constructed: one representing fresh ginger and the other representing ginger dried at 65 °C in a hot-air oven for 2 h. The recombinants were prepared by blending the previously identified key odorants (OAV ≥ 1) at their naturally occurring concentrations in an odorless matrix. Sensory evaluation was performed on two pairs: sample 1 (SAFE extract) was compared with its recombinant, and sample 5 (SAFE extract) was compared with its recombinant. A trained panel of 10 assessors conducted QDA to compare each recombinant with its corresponding original extract. As shown in the radar plots (Figure 4), the mean aroma profiles of both recombinant samples closely matched those of their original counterparts, and no statistically significant differences were detected for any attribute (paired *t*-tests, *p* > 0.05), thereby supporting the conclusion that the previously identified odorants are largely sufficient to reconstitute the characteristic aromas of both fresh and dried ginger.

## 4. Conclusions

This study systematically characterized the dynamic changes in the aroma profile of ginger (from Weifang City, Shandong Province, China) during hot-air drying. Sensory evaluation demonstrated a progressive shift from the characteristic pungent, floral, and herbal notes of fresh ginger toward predominantly roasted and woody attributes as drying progressed, with a transient enhancement of sweetness observed at the intermediate stage (1.0 h). Instrumental analysis identified 92 volatile compounds, among which terpenes exhibited a marked increase in absolute concentration in response to drying. Through the integrated application of AEDA, OAV calculation, and aroma-recombination experiments, 19 key odor-active compounds were confirmed to be the primary contributors to the characteristic aroma of ginger.

These findings establish a quantitative molecular basis for the drying-induced aroma modulation in ginger and offer practical guidance for optimizing postharvest processing to achieve desired sensory qualities in ginger products. However, this study also has certain limitations. First, this study selected only ginger samples from Weifang, Shandong, for experimentation and analysis. Therefore, whether the effects of hot-air drying on flavor compounds are universal across different ginger varieties requires further verification through an expanded sample size. Second, in terms of experimental methods, comprehensive two-dimensional gas chromatography with high peak capacity, high resolution, and high sensitivity can be used to obtain more precise fingerprint profiles. Third, the lack of quantification of pungent non-volatiles (gingerols and shogaols) and the use of only a single drying temperature (65 °C) leave the integration of pungency with aroma and the impacts of multi-temperature or staged drying strategies unexplored. In the future, more in-depth research on ginger drying processes could be conducted from the perspectives outlined above.

## Figures and Tables

**Table 1:** Changes in the content of volatile compounds in ginger during the roasting process.

Type	No.	Compounds	CAS	RI	Content (μg/g)				
					1	2	3	4	5
Alcohols	1	2-Heptanol	543-49-7	1328	6.61 ± 1.66 a	5.11 ± 0.59 a	4.75 ± 2.74 a	0.48 ± 0.09 b	0.88 ± 0.05 b
	2	2-Nonanol	628-99-9	1524	1.72 ± 0.43 b	2.31 ± 0.22 ab	3.28 ± 1.76 a	0.09 ± 0.02 c	0.84 ± 0.14 bc
	3	Linalool	78-70-6	1552	15.19 ± 2.07 a	7.56 ± 0.85 b	8.45 ± 2.38 b	ND	ND
	4	Terpinen-4-ol	562-74-3	1596	6.48 ± 0.70	ND	ND	1.92 ± 0.12	ND
	5	Citronellol	106-22-9	1769	1.57 ± 0.05 a	0.40 ± 0.02 b	ND	0.07 ± 0.01 d	0.17 ± 0.06 c
	6	Myrtenol	515-00-4	1783	0.41 ± 0.05 a	0.23 ± 0.04 b	0.39 ± 0.07 a	0.16 ± 0.02 bc	0.12 ± 0.01 c
	7	Nerol	106-25-2	1797	0.27 ± 0.24	ND	ND	ND	ND
	8	Geraniol	106-24-1	1848	15.45 ± 3.57 a	0.64 ± 0.12 b	0.81 ± 0.16 b	0.46 ± 0.12 b	0.25 ± 0.06 b
	9	(*E*)-Nerolidol	40716-66-3	2040	11.09 ± 2.26 a	3.61 ± 0.45 bc	4.88 ± 0.88 b	2.63 ± 0.33 c	2.37 ± 0.14 c
	10	*α*-Elemol	639-99-6	2067	2.99 ± 0.70 a	1.05 ± 0.10 c	1.49 ± 0.17 bc	1.38 ± 0.24 bc	1.82 ± 0.23 b
	11	*γ*-Eudesmol	1209-71-8	2194	2.03 ± 0.41 a	0.63 ± 0.06 b	0.86 ± 0.16 b	0.62 ± 0.18 b	0.18 ± 0.03 c
	12	*β*-Eudesmol	473-15-4	2200	5.98 ± 1.20 a	2.74 ± 0.35 bc	3.41 ± 0.64 b	1.99 ± 0.21 c	1.66 ± 0.13 c
	13	*α*-Cadinol	481-34-5	2210	0.56 ± 0.12 a	0.27 ± 0.04 bc	0.32 ± 0.06 b	0.15 ± 0.02 d	0.17 ± 0.01 cd
	14	Spathulenol	6750-60-3	2286	1.07 ± 0.13 a	0.63 ± 0.05 bc	0.71 ± 0.17 b	0.46 ± 0.01 c	0.52 ± 0.05 c
	15	Cedrenol	28231-03-0	2347	2.08 ± 0.57 a	0.99 ± 0.06 b	1.12 ± 0.20 b	0.93 ± 0.10 b	2.63 ± 0.90 a
	16	Geranyl linalool	1113-21-9	2501	0.77 ± 0.24 a	0.29 ± 0.06 b	0.51 ± 0.16 b	0.52 ± 0.03 ab	0.50 ± 0.02 b
	17	Zingiberenol	58334-55-7	2100	11.78 ± 1.97 a	5.80 ± 0.18 c	7.78 ± 1.22 b	5.15 ± 0.71 d	4.33 ± 0.28 d
	18	*α*-Terpineol	98-55-5	1766	47.10 ± 6.39	ND	ND	ND	7.12 ± 8.94
	19	endo-Borneol	507-70-0	1691	71.25 ± 23.06 a	18.54 ± 6.31 b	18.06 ± 15.73 b	8.52 ± 7.62 b	ND
	20	(1R,4S,5R,6R,7S,10R)-7-isopropyl-4,10-dimethyltricyclo [4.4.0.01,5]decan-4-ol	23445-02-5	1925	1.43 ± 0.09 a	0.88 ± 0.06 b	1.05 ± 0.21 b	0.57 ± 0.05 c	0.58 ± 0.03 c
	21	*β*-Bisabolol	15352-77-9	2136	1.03 ± 0.26 a	0.33 ± 0.01 b	0.38 ± 0.07 b	0.27 ± 0.04 b	0.21 ± 0.02 b
	22	*p*-Cymen-8-ol	1197-01-9	1844	ND	ND	0.21 ± 0.05	0.06 ± 0.01	0.05 ± 0.00
Subtotal					206.86 ± 46.17	52.01 ± 9.57	58.46 ± 26.83	26.43 ± 9.93	24.40 ± 11.13
Aldehydes	23	Octanal	124-13-0	1292	1.11 ± 0.20 a	0.37 ± 0.05 bc	0.40 ± 0.14 b	0.19 ± 0.01 cd	0.17 ± 0.01 d
	24	(*E*)-2-Octenal	2548-87-0	1429	1.07 ± 0.15 a	0.44 ± 0.05 b	0.47 ± 0.11 b	0.19 ± 0.01 c	0.21 ± 0.01 c
	25	Benzaldehyde	100-52-7	1517	0.20 ± 0.08	ND	ND	ND	0.13 ± 0.02
	26	Myrtenal	564-94-3	1611	0.83 ± 0.15 a	0.55 ± 0.03 b	0.58 ± 0.11 b	0.22 ± 0.04 c	0.24 ± 0.03 c
	27	(*E*)-2-Decenal	3913-81-3	1639	0.65 ± 0.10 a	0.55 ± 0.02 a	ND	0.17 ± 0.02 b	0.21 ± 0.05 b
	28	Neral	106-26-3	1675	48.74 ± 10.89 a	31.45 ± 1.54 b	37.80 ± 14.95 ab	11.81 ± 1.63 c	11.90 ± 1.81 c
	29	Geranial	141-27-5	1728	129.63 ± 23.57 a	66.95 ± 5.88 b	70.57 ± 26.09 b	29.89 ± 2.39 c	24.25 ± 3.09 c
	30	Ethyl citral	41448-29-7	2406	2.35 ± 0.37	ND	ND	ND	ND
	31	Cinnamaldehyde	104-55-2	2019	ND	ND	ND	ND	0.10 ± 0.02
	32	2,6-Dimethyl-5-heptenal	106-72-9	1416	0.14 ± 0.03	ND	ND	ND	0.04 ± 0.01
	33	Citronellal	106-23-0	1542	3.18 ± 0.43	ND	ND	ND	1.29 ± 0.21
	34	Decanal	112-31-2	1563	ND	ND	ND	ND	0.51 ± 0.81
	35	(*E*)-2-Dodecenal	20407-84-5	1931	0.40 ± 0.11	ND	ND	ND	0.12 ± 0.02
	36	Vanillin	121-33-5	2547	0.25 ± 0.01 a	0.08 ± 0.06 b	ND	0.12 ± 0.01 b	0.11 ± 0.02 b
Subtotal					188.55 ± 36.09	100.39 ± 7.63	109.82 ± 41.40	42.59 ± 4.11	39.28 ± 6.11
Terpenes	37	Sabinene	3387-41-5	1124	2.35 ± 0.30 a	1.67 ± 0.14 b	1.52 ± 0.26 b	0.61 ± 0.02 c	0.67 ± 0.13 c
	38	1,3,8-*p*-Menthatriene	18368-95-1	1155	0.82 ± 0.19 a	0.32 ± 0.05 b	0.25 ± 0.11 bc	0.11 ± 0.01 c	ND
	39	*α*-Phellandrene	99-83-2	1167	4.82 ± 0.58 a	4.32 ± 1.09 ab	4.15 ± 2.04 ab	1.78 ± 0.24 c	2.60 ± 0.57 bc
	40	Myrcene	123-35-3	1170	38.27 ± 7.96 a	20.49 ± 3.53 b	17.01 ± 4.89 b	6.65 ± 0.22 c	6.07 ± 0.66 c
	41	*α*-Terpinene	99-86-5	1180	0.61 ± 0.10	0.34 ± 0.03	0.29 ± 0.06	0.13 ± 0.01	0.13 ± 0.03
	42	Limonene	5989-27-5	1197	36.93 ± 4.55 a	21.98 ± 1.80 b	20.35 ± 3.97 b	9.39 ± 0.35 c	8.96 ± 1.37 c
	43	(*E*)-*β*-Ocimene	3779-61-1	1242	0.11 ± 0.01	ND	ND	ND	ND
	44	*γ*-Terpinene	99-85-4	1247	0.82 ± 0.05 a	0.42 ± 0.04 b	0.40 ± 0.15 b	0.21 ± 0.03 c	0.18 ± 0.01 c
	45	Terpinolene	586-62-9	1281	6.22 ± 0.64	3.05 ± 0.24	3.15 ± 1.23	1.53 ± 0.07	1.25 ± 0.07
	46	Copaene	3856-25-5	1481	14.53 ± 1.66 a	9.41 ± 0.77 b	11.86 ± 3.51 ab	5.71 ± 0.56 c	5.49 ± 0.52 c
	47	*α*-Cubebene	17699-14-8	1450	ND	0.11 ± 0.00	0.16 ± 0.06	ND	0.09 ± 0.01
	48	Cubebene	13744-15-5	1528	1.41 ± 0.20 a	1.28 ± 0.03 a	ND	0.64 ± 0.05 b	0.77 ± 0.06 b
	49	*β*-Elemene	515-13-9	1580	7.06 ± 1.17 a	3.51 ± 0.31 b	4.40 ± 1.24 b	3.24 ± 0.73 b	4.78 ± 0.33 b
	50	Alloaromadendrene	25246-27-9	1626	5.65 ± 0.66	3.31 ± 0.30	3.94 ± 1.15	2.02 ± 0.24	2.37 ± 0.15
	51	*β*-Sesquiphellandrene	20307-83-9	1761	116.03 ± 10.96 a	74.10 ± 6.40 c	93.17 ± 18.47 b	57.67 ± 3.22 cd	53.29 ± 4.45 d
	52	*γ*-Selinene	515-17-3	1660	3.72 ± 0.46 a	2.29 ± 0.07 b	ND	ND	1.38 ± 0.16 c
	53	(*E*)-*β*-Farnesene	18794-84-8	1666	10.63 ± 1.23 a	5.61 ± 0.33 b	6.83 ± 1.70 b	3.95 ± 0.36 c	3.37 ± 0.08 c
	54	Germacrene D	23986-74-5	1688	ND	23.99 ± 3.36	29.36 ± 10.97	17.56 ± 9.10	ND
	55	*β*-Selinene	17066-67-0	1702	14.32 ± 0.89 a	10.52 ± 0.25 b	12.39 ± 2.24 ab	6.79 ± 0.86 c	7.58 ± 0.70 c
	56	Zingiberene	495-60-3	1717	467.56 ± 59.46 a	314.05 ± 17.05 b	405.96 ± 66.98 a	244.28 ± 22.23 bc	214.97 ± 22.14 c
	57	*β*-Bisabolene	495-61-4	1721	73.74 ± 4.64 a	45.63 ± 1.73 c	55.06 ± 9.83 b	32.51 ± 2.70 d	31.34 ± 1.95 d
	58	*α*-Farnesene	502-61-4	1749	81.52 ± 12.10 a	44.59 ± 2.50 b	49.50 ± 13.25 b	19.47 ± 1.31 c	21.02 ± 1.88 c
	59	*α*-Curcumene	644-30-4	1765	48.58 ± 5.49 a	38.55 ± 1.37 b	42.89 ± 5.39 ab	21.19 ± 1.76 c	20.68 ± 3.19 c
	60	*β*-Phellandrene	555-10-2	1204	ND	116.83 ± 2.55	116.15 ± 17.50	47.99 ± 1.99	52.14 ± 7.77
	61	(*E*)-Bergamotene	13474-59-4	1574	ND	0.78 ± 0.06	0.76 ± 0.14	ND	ND
	62	*γ*-Gurjunene	22567-17-5	1740	ND	6.74 ± 0.35	7.88 ± 2.09	3.65 ± 0.21	ND
	63	*α*-Pinene	80-56-8	1121	42.94 ± 6.13	ND	ND	ND	8.13 ± 0.24
	64	*β*-Pinene	127-91-3	1182	8.27 ± 1.54	ND	ND	ND	0.96 ± 0.03
	65	*γ*-Cadinene	39029-41-9	1696	35.75 ± 1.61 a	20.62 ± 1.44 b	24.04 ± 6.05 b	11.97 ± 1.59 c	13.92 ± 0.18 c
	66	Caryophyllene oxide	1139-30-6	1996	0.31 ± 0.08	0.15 ± 0.02	ND	ND	ND
	67	Limonene oxide	1195-92-2	1977	0.23 ± 0.02	0.43 ± 0.17	0.48 ± 0.16	ND	ND
	68	*δ*-Elemene	20307-84-0	1459	ND	0.08 ± 0.01 b	0.10 ± 0.01 a	0.05 ± 0.00 c	0.06 ± 0.01 c
	69	*γ*-Muurolene	30021-74-0	1680	ND	1.33 ± 0.08	1.76 ± 0.75	ND	ND
Subtotal					1023.20 ± 122.68	777.83 ± 46.15	915.57 ± 174.95	499.10 ± 47.86	462.20 ± 46.69
Aromatic hydrocarbons	70	Ethylbenzene	100-41-4	1132	2.71 ± 0.45	0.70 ± 0.13	ND	ND	ND
	71	*p*-Xylene	106-42-3	1140	3.22 ± 0.57 a	1.72 ± 0.29 b	0.91 ± 0.04 c	0.83 ± 0.22 c	ND
	72	*o*-Xylene	95-47-6	1146	ND	1.22 ± 0.17 b	1.38 ± 0.07 b	1.16 ± 0.32 b	1.88 ± 0.11 a
	73	*m*-Xylene	108-38-3	1131	ND	ND	0.81 ± 0.06	0.70 ± 0.17	1.06 ± 0.09
	74	*p*-*α*-Dimethylstyrene	1195-32-0	1436	0.17 ± 0.02 a	0.09 ± 0.01 b	0.09 ± 0.02 b	ND	0.05 ± 0.00 c
	75	Styrene	100-42-5	1259	ND	ND	ND	ND	0.79 ± 0.06
Subtotal					6.10 ± 1.04	3.73 ± 0.60	3.19 ± 0.19	2.69 ± 0.71	3.78 ± 0.26
Ketones	76	6-Methyl-5-hepten-2-one	110-93-0	1342	2.75 ± 1.76 a	0.70 ± 0.13 b	0.69 ± 0.14 b	0.17 ± 0.03 b	0.21 ± 0.02 b
	77	2-Nonanone	821-55-6	1390	0.76 ± 0.21 b	1.24 ± 0.10 a	1.23 ± 0.50 a	0.18 ± 0.01 c	0.57 ± 0.09 bc
	78	Vanillylacetone	122-48-5	2778	1.54 ± 0.72 a	0.68 ± 0.10 b	0.52 ± 0.08 b	0.78 ± 0.05 b	0.91 ± 0.02 ab
	79	Irisone	14901-07-6	1971	0.28 ± 0.06 a	0.13 ± 0.02 b	0.15 ± 0.06 b	0.10 ± 0.05 b	0.08 ± 0.00 b
	80	2-Undecanone	112-12-9	1595	ND	5.78 ± 0.37	6.86 ± 1.79	ND	3.53 ± 0.37
	81	Camphor	464-48-2	1498	2.85 ± 0.39 a	1.15 ± 0.14 b	1.24 ± 0.45 b	0.52 ± 0.02 c	0.44 ± 0.08 c
Subtotal					8.18 ± 3.14	9.68 ± 0.86	10.69 ± 3.02	1.75 ± 0.16	5.74 ± 0.58
Carboxylic acids	82	Lauric acid	143-07-7	2485	1.35 ± 0.44 a	0.46 ± 0.07 b	0.38 ± 0.19 b	0.41 ± 0.13 b	0.34 ± 0.04 b
	83	Octanoic acid	124-07-2	2060	0.27 ± 0.12	ND	ND	ND	ND
Subtotal					1.62 ± 0.56	0.46 ± 0.07	0.38 ± 0.19	0.41 ± 0.13	0.34 ± 0.04
Ester	84	4,8-Dimethyl-3,7-nonadien-2-ylacetate	91418-25-6	1310	0.82 ± 0.12	ND	ND	ND	ND
	85	Methyl hexanoate	106-70-7	1191	ND	ND	ND	ND	0.05 ± 0.01
	86	Bornyl acetate	76-49-3	1569	4.03 ± 0.73 a	1.18 ± 0.11 b	1.31 ± 0.75 b	0.77 ± 0.06 b	0.54 ± 0.04 b
Subtotal					4.85 ± 0.85	1.18 ± 0.11	1.31 ± 0.75	0.77 ± 0.06	0.59 ± 0.05
Ether	87	(*Z*)-Linalool oxide	5989-33-3	1441	0.48 ± 0.12 a	0.14 ± 0.02 b	0.12 ± 0.06 b	0.07 ± 0.01 b	ND
	88	Methyl isoeugenol	93-16-3	2175	1.13 ± 0.22 a	0.14 ± 0.06 b	0.15 ± 0.03 b	0.11 ± 0.01 b	0.11 ± 0.02 b
	89	1,8-Cineole	470-82-6	1205	183.86 ± 19.01	ND	ND	ND	ND
Subtotal					185.47 ± 19.35	0.28 ± 0.08	0.27 ± 0.09	0.18 ± 0.02	0.11 ± 0.02
Others	90	Rosefuran	15186-51-3	1403	0.52 ± 0.22 a	0.26 ± 0.03 bc	0.37 ± 0.14 ab	0.13 ± 0.00 c	0.12 ± 0.02 c
	91	Perillen	539-52-6	1421	1.00 ± 0.28 a	0.35 ± 0.04 b	0.37 ± 0.18 b	0.15 ± 0.03 b	0.15 ± 0.02 b
	92	2-Acetyl pyrrole	1072-83-9	1959	ND	ND	ND	0.11 ± 0.05	0.13 ± 0.01
Subtotal					1.52 ± 0.50	0.61 ± 0.07	0.74 ± 0.32	0.39 ± 0.08	0.40 ± 0.05

“ND”, NOT Detected. Note: Different letters in each row indicate significance (*p* < 0.05).

**Table 2:** Odor-active compounds determined by AEDA in samples 1 and 5.

No.	Compounds	CAS	Odorant Description ^a^	RI ^b^	FD Factor		Identification Method
					1	5	
1	1,8-Cineole	470-82-6	Herbal, Camphor, Medicinal	1205	8	/	MS, RI, STD, O
2	2-Heptanol	543-49-7	Lemon, Grass, Herbal, Sweet, Floral	1328	64	32	MS, RI, STD, O
3	2-Nonanol	628-99-9	Waxy, Green, Creamy, Citrus, Fruity	1524	64	8	MS, RI, STD, O
4	Linalool	78-70-6	Citrus, Floral, Sweet, Woody, Green	1552	512	512	MS, RI, STD, O
5	Geraniol	106-24-1	Sweet, Floral, Fruity, Waxy, Citrus	1848	1024	512	MS, RI, STD, O
6	(*E*)-Nerolidol	40716-66-3	Floral, Green, Citrus, Woody, Waxy	2040	256	32	MS, RI, STD, O
7	*β*-Eudesmol	473-15-4	Woody	2200	4	64	MS, RI, STD, O
8	Spathulenol	6750-60-3	Herbal, Fruity	2286	2	/	MS, RI, O
9	Cedrenol	28231-03-0	Woody	2347	16	16	MS, RI, O
10	Geranyl linalool	1113-21-9	Mild, Fatty, Coconut, Oil	2659	8	/	MS, RI, STD, O
11	*α*-Cadinol	481-34-5	Spicy, Herbal, Green Tea	2210	/	16	MS, RI, O
12	*α*-Terpineol	98-55-5	Woody, Floral	1766	256	32	MS, STD, O
13	(1R,4S,5R,6R,7S,10R)-7-isopropyl-4,10-dimethyltricyclo [4.4.0.01,5]decan-4-ol	23445-02-5	Spicy, Green	1999	32	/	MS, O
14	*α*-Pinene	80-56-8	Fresh, Camphor, Earthy, Woody	1120	2	2	MS, STD, O
15	*β*-Pinene	127-91-3	Herbal	1182	4	4	MS, STD, O
16	Myrcene	123-35-3	Peppery, Terpene, Spicy, Balsam, Plastic	1170	256	64	MS, RI, STD, O
17	*γ*-Terpinene	99-85-4	Oily, Woody, Lemon, Herbal	1247	8	16	MS, RI, STD, O
18	Copaene	3856-25-5	Woody, Spicy, Honey	1481	256	64	MS, RI, O
19	Zingiberene	495-60-3	Spice, Fresh, Sharp,	1716	1024	512	MS, RI, STD, O
20	*β*-Bisabolene	495-61-4	Woody	1721	2	2	MS, RI, O
21	*α*-Farnesene	502-61-4	Citrus, Herbal, Lavender, Bergamo	1749	32	64	MS, RI, STD, O
22	*α*-Curcumene	644-30-4	Herbal	1765	4	16	MS, RI, O
23	Cadina-1,3,5-triene	483-77-2	Vanilla	1812	8	/	MS, O
24	Limonene	5989-27-5	Citrus, Orange, Fresh, Sweet	1197	/	8	MS, RI, STD, O
25	Sabinene	3387-41-5	Woody, Terpene, Citrus, Pine, Spice	1124	/	4	MS, RI, STD, O
26	*α*-Cubebene	17699-14-8	Herbal, Waxy	1451	/	512	MS, RI, O
27	*β*-Elemene	515-13-9	Sweet	1580	8	8	MS, RI, STD, O
28	(*E*)-*β*-Farnesene	18794-84-8	Woody, Citrus, Herbal, Sweet	1666	/	16	MS, RI, STD, O
29	*β*-Phellandrene	555-10-2	Mint	1204	/	8	MS, RI, STD, O
30	3-Ethoxy-3,7-dimethyl-1,6-octadiene	72845-33-1	Floral	2627	4	/	MS, O
31	Octanal	124-13-0	Waxy, Citrus, Herbal, Fresh, Fatty	1291	64	256	MS, RI, STD, O
32	(*E*)-2-Octenal	2548-87-0	Cucumber, Fatty, Herbal, Banana	1429	4	/	MS, RI, STD, O
33	Myrtenal	564-94-3	Sweet, Spicy, Terpene, Camphor, Jam	1611	32	128	MS, RI, O
34	Neral	106-26-3	Sweet, Lemon	1675	64	256	MS, RI, STD, O
35	(*E*)-2-Dodecenal	20407-84-5	Spicy, Woody, Bitter, Coffee, Citrus	1931	1024	128	MS, STD, O
36	(*E,Z*)-2,6-Dodecadienal	21662-13-5	Citrus	1955	256	32	MS, STD, O
37	Citronellal	106-23-0	Sweet, Floral, Herbal	1542	64	1024	MS, STD, O
38	Decanal	112-31-2	Sweet, Waxy, Orange Peel, Citrus, Floral	1563	/	2	MS, STD, O
39	Cinnamaldehyde	104-55-2	Sweet, Spicy, Resinous, Honey	2019	/	128	MS, RI, STD, O
40	(*E*)-2-Decenal	3913-81-3	Waxy, Fatty, Earthy, Green, Coriander	1639	/	128	MS, RI, STD, O
41	2,6-Dimethyl-5-heptenal	106-72-9	Sweet, Fruity, Minty, Fresh, Floral	1416	256	16	MS, STD, O
42	*o*-Xylene	95-47-6	Geranium	1146	4	/	MS, RI, STD, O
43	*m*-Xylene	108-38-3	Geranium	1130	/	4	MS, RI, STD, O
44	6-Methyl-5-hepten-2-one	110-93-0	Citrus, Musty, Lemongrass, Apple	1342	16	16	MS, RI, STD, O
45	2-Nonanone	821-55-6	Sweet, Green, Weedy, Earthy, Herbal	1389	16	2	MS, RI, STD, O
46	Linalyl formate	115-99-1	Citrus, Woody	1317	/	16	MS, O
47	(*Z*)-Linalool oxide	5989-33-3	Earthy, Floral, Woody	1502	256	4	MS, O
48	Camphor	464-48-2	Camphor	1498	64	/	MS, RI, STD, O
49	Bornyl acetate	76-49-3	Woody, Pine, Herbal, Cedar, Spice	1568	16	4	MS, RI, STD, O
50	2-Acetylpyrrole	1072-83-9	Musty, Nut, Maraschino, Cherry, Walnut	1958	/	16	MS, RI, STD, O
51	Caryophyllene oxide	1139-30-6	Sweet, Fresh, Dry, Woody, Spicy	1996	8	32	MS, RI, STD, O
52	*o*-Cresol	95-48-7	Moldy, Plastic, Medicinal Herbal	2127	128	/	MS, RI, O
53	Vanillin	121-33-5	Vanilla	2547	32	8	MS, RI, STD, O
54	Lauric acid	143-07-7	Mild, Fatty, Coconut, Bay, Oil	2485	/	128	MS, RI, STD, O
55	Rosefuran	15186-51-3	Spearmint, Creamy, Sweet	1467	/	4	MS, O

^a^ Odor quality perceived at the sniffing port. ^b^ RI; compounds were identified on a DB-WAX. “/” indicates not found in the AEDA.

**Table 3:** Quantitative results, odor threshold, and OAVs of key flavor substances in samples 1 and 5.

No.	Compounds	CAS	Standard Curves	R^2^	Content (μg/g)		Odor Threshold (mg/kg) ^a^	OAV	
					1	5		1	5
1	Myrcene	123-35-3	y = 0.5606x + 0.1172	0.9972	66.10 ± 14.20	8.66 ± 1.17	0.10	661.00	86.60
2	1,8-Cineole	470-82-6	y = 0.181x + 0.1556	0.9992	1006.89 ± 105.01	0.00	1.00	1006.89	0.00
3	Limonene	5989-27-5	y = 0.4879x + 0.2333	0.9980	70.72 ± 9.32	13.40 ± 2.82	1.20	58.93	11.17
4	*γ*-Terpinene	99-85-4	y = 0.7003x − 0.0012	0.9928	1.19 ± 0.07	0.27 ± 0.02	1.00	1.19	0.27
5	Octanal	124-13-0	y = 0.3436x + 0.0029	0.9931	3.14 ± 0.57	0.40 ± 0.02	0.01	314.00	40.00
6	2-Heptanol	543-49-7	y = 0.632x + 0.0281	0.9983	10.00 ± 2.63	0.93 ± 0.08	0.04	250.00	23.25
7	6-Methyl-5-hepten-2-one	110-93-0	y = 0.2022x + 0.0131	0.9973	12.95 ± 8.71	0.36 ± 0.09	0.16	80.94	2.25
8	2-Nonanone	821-55-6	y = 0.9695x − 0.0058	0.9978	0.84 ± 0.21	0.65 ± 0.09	0.02	42.00	32.50
9	Camphor	464-48-2	y = 4.6506x + 0.0185	0.9953	0.57 ± 0.08	0.05 ± 0.02	1.47	0.39	0.03
10	2-Nonanol	628-99-9	y = 1.4002x − 0.0005	0.9979	1.23 ± 0.31	0.60 ± 0.10	0.09	13.67	6.67
11	Linalool	78-70-6	y = 0.3603x + 0.0688	0.9905	40.17 ± 5.75	0.00	0.01	4017.00	0.00
12	Bornyl acetate	76-49-3	y = 0.4486x + 0.0107	0.9983	8.74 ± 1.62	0.95 ± 0.09	1.38	6.30	0.69
13	*β*-Elemene	515-13-9	y = 0.3855x − 0.024	0.9981	18.97 ± 3.04	13.04 ± 0.85	/	/	/
14	Myrtenal	564-94-3	y = 0.1026x + 0.0111	0.9987	6.96 ± 1.45	1.24 ± 0.28	/	/	/
15	*(E*)-2-Decenal	3913-81-3	y = 0.192x − 0.0024	0.9999	3.52 ± 0.54	1.22 ± 0.26	0.02	176.00	61.00
16	(*E*)-*β*-Farnesene	18794-84-8	y = 1.4326x + 0.0671	0.9985	6.93 ± 0.86	1.86 ± 0.05	/	/	/
17	Neral	106-26-3	y = 0.1968x + 0.1176	0.9934	241.47 ± 55.33	54.25 ± 9.21	0.10	2414.70	542.50
18	Zingiberene	495-60-3	y = 0.2574x + 0.6595	0.9928	1789.87 ± 230.99	808.58 ± 86.01	/	/	/
19	*α*-Farnesene	502-61-4	y = 0.0946x + 0.0046	0.9967	861.27 ± 127.94	221.65 ± 19.89	/	/	/
20	Geraniol	106-24-1	y = 0.5974x + 0.0106	0.9984	25.68 ± 5.98	0.24 ± 0.10	0.01	2568.00	24.00
21	2-Acetylpyrrole	1072-83-9	y = 0.6692x − 0.0105	0.9992	0.00	0.36 ± 0.02	58.00	0.00	0.01
22	Cinnamaldehyde	104-55-2	y = 5.4798x − 0.0081	0.9999	0.00	0.03 ± 0.00	0.75	0.00	0.04
23	Caryophyllene oxide	1139-30-6	y = 0.3259x + 0.0118	0.9935	0.58 ± 0.24	0.00	0.41	1.41	/
24	(*E*)-Nerolidol	40716-66-3	y = 0.2442x + 0.0035	0.9991	45.25 ± 9.26	9.56 ± 0.59	0.25	181.00	38.24
25	Lauric acid	143-07-7	y = 0.2924x − 0.0067	0.9999	4.85 ± 1.49	1.39 ± 0.13	/	/	/
26	Geranyllinalool	1113-21-9	y = 0.306x + 0.061	0.9946	1.92 ± 0.86	1.78 ± 0.19	/	/	/
27	Vanillin	121-33-5	y = 0.4217x − 0.0157	0.9992	0.98 ± 0.01	0.65 ± 0.04	0.03	32.67	21.67
28	2,6-Dimethyl-5-heptenal	106-72-9	y = 0.9955x − 0.0155	0.9985	0.31 ± 0.03	0.20 ± 0.01	0.02	15.50	10.00
29	Citronellal	106-23-0	y = 1.3878x − 0.0052	0.9939	2.33 ± 0.31	0.97 ± 0.15	0.03	77.67	32.34
30	*α*-Terpineol	98-55-5	y = 1.0905x − 0.0195	0.9986	43.38 ± 5.86	9.73 ± 0.99	0.28	154.93	34.75
31	(*E*)-2-Dodecenal	20407-84-5	y = 0.4985x − 0.0119	0.9971	1.05 ± 0.22	0.49 ± 0.04	0.01	105.00	49.00

^a^ Odor thresholds in water are from reference [15]. “/” indicates not found in the literature.

## Data Availability

The original contributions presented in this study are included in the article/Supplementary Materials. Further inquiries can be directed to the corresponding author.

## Supplementary Materials

The following supporting information can be downloaded at: https://www.mdpi.com/article/10.3390/foods15132377/s1. Table S1. Drying parameters of ginger in the drying process. Figure S1. Residual Plot of the Analytes Listed in Table 3.

## Abbreviations

The following abbreviations are used in the manuscript:

VOCs	Volatile organic compounds
SAFE	Solvent-assisted flavor evaporation
GC-MS	Gas chromatography–mass spectrometry
GC-O-MS	Gas chromatography–olfactometry–mass spectrometry
AEDA	Aroma extract dilution analysis
OAV	Odor activity value
QDA	Quantitative descriptive analysis
EI	Electron ionization
FD	Flavor dilution
RIs	Retention indexes
SIM	Selected ion monitoring
SD	Standard deviation
ANOVA	Analysis of variance
PCA	Principal component analysis
OPLS-DA	Orthogonal partial least squares-discriminant analysis
HCA	Hierarchical cluster analysis
VIP	Variable importance in projection
