# Supplementary material for: Characterization of the Flavor Profiles of Fresh and Hot-Air-Dried Ginger (Zingiber officinale Roscoe) by Molecular Sensory Science
Source: Foods. 2026 Jul 3;15(13):2377. doi: 10.3390/foods15132377 (PMC13362381; doi:10.3390/foods15132377)
Supplement: Supplementary file 1 [file foods-15-02377-s001.zip › foods-4369005-supplementary.pdf]

Table S1. Drying parameters of ginger in the drying process.

Sample	1	2	3	4	5	Control Group
Oven temperature (°C)	0	65	65	65	65	65
Drying time (h)	0	0.5	1	1.5	2	7.5
Add ginger (g)	40	40	40	40	40	40
Output ginger (g)	40	20	5	2.6	2.56	2.267
Sample mass for SAFE (g)	40	18.1	4.93	2.56	2.43	/
Moisture content (%)	95.2	88.7	57.2	13.5	11.6	Dry basis

"/" indicates that the control group did not undergo SAFE.

Figure S1. Residual Plot of the Analytes Listed in Table 3

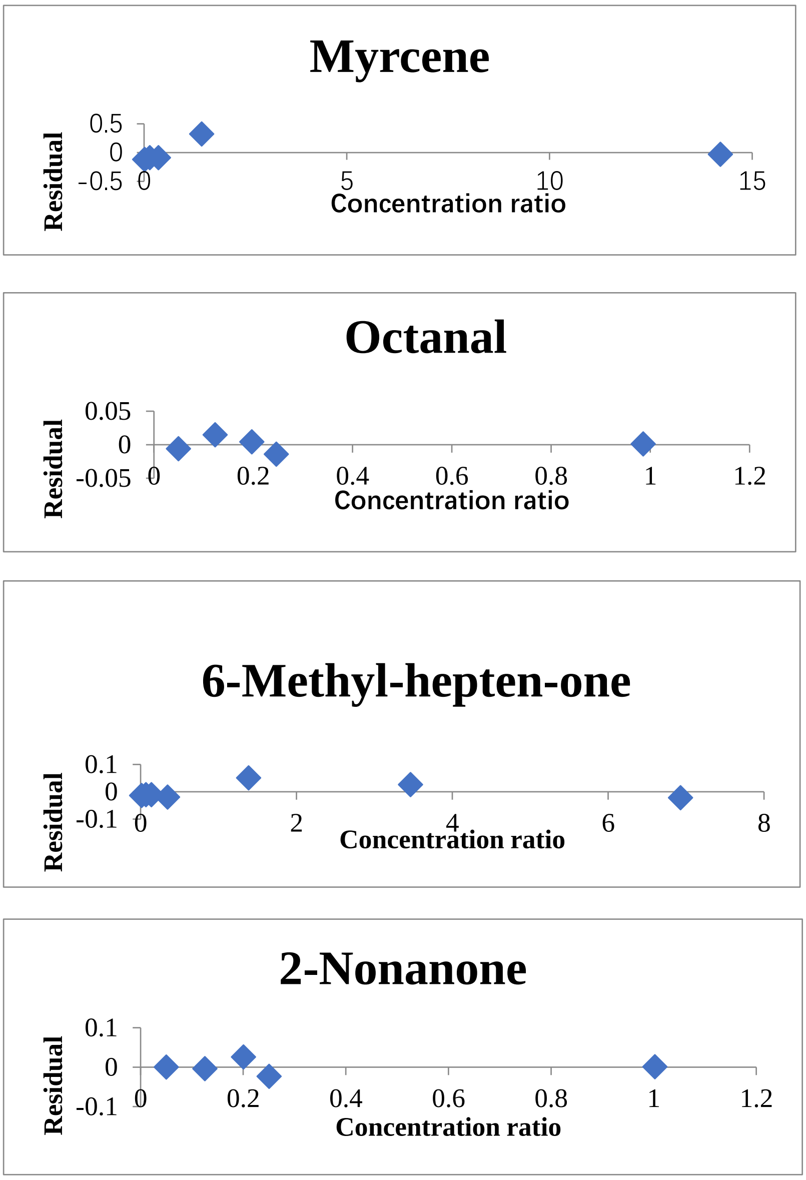

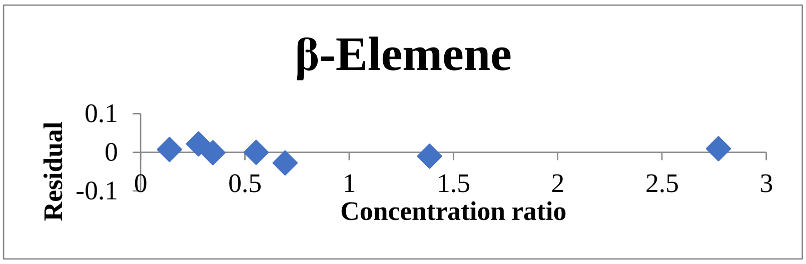
β-Elemene
Residual
0.1
0
-0.1
0
0.5
1
1.5
2
2.5
3
Concentration ratio

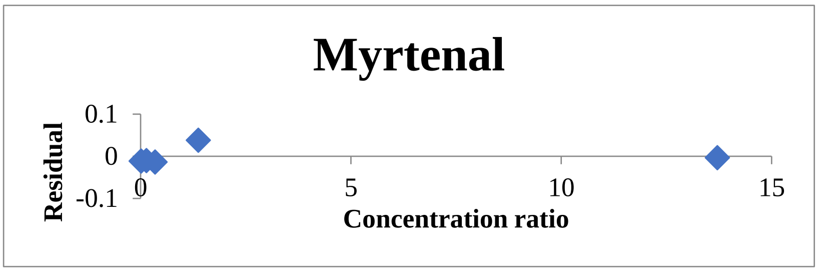
Myrtenal
Residual
0.1
0
-0.1
0
5
10
15
Concentration ratio

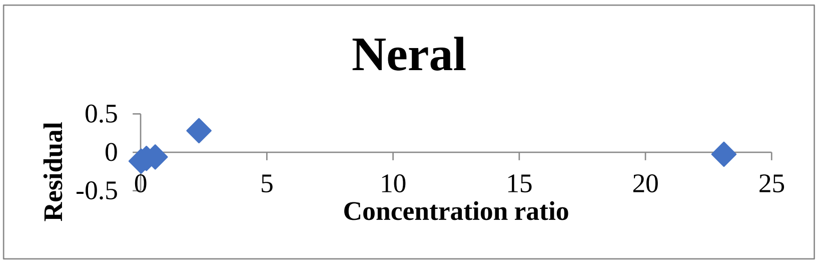
Neral
Residual
0.5
0
-0.5
0
5
10
15
20
25
Concentration ratio

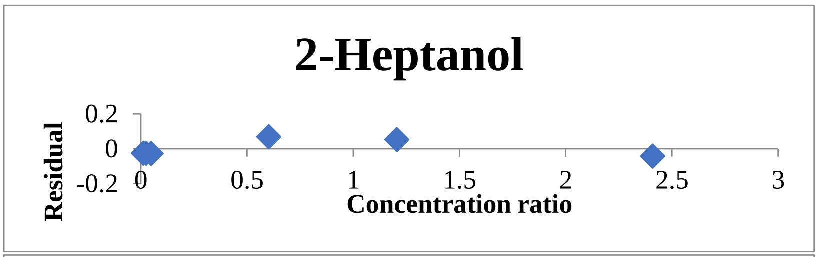
2-Heptanol
Residual
0.2
0
-0.2
0
0.5
1
1.5
2
2.5
3
Concentration ratio

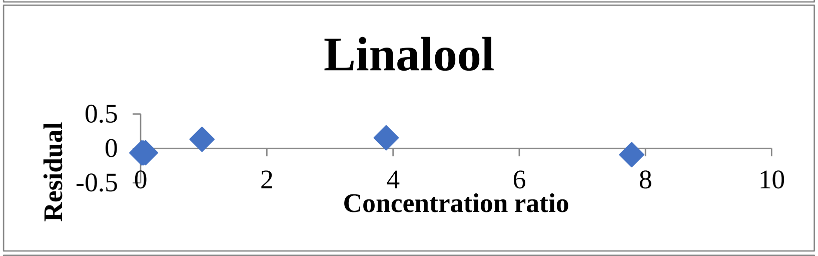
Linalool
Residual
0.5
0
-0.5
0
2
4
6
8
10
Concentration ratio

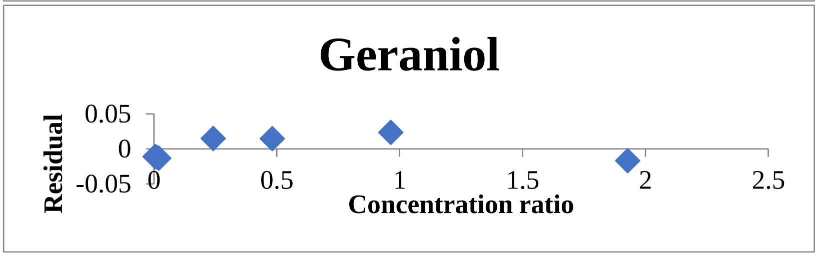
Geraniol
Residual
0.05
0
-0.05
0
0.5
1
1.5
2
2.5
Concentration ratio

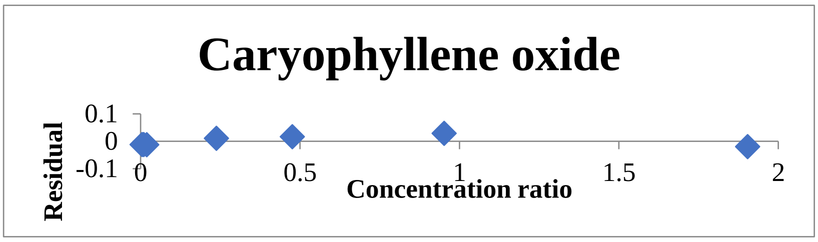
Caryophyllene oxide
Residual
0.1
0
-0.1
0
0.5
1
1.5
2
Concentration ratio

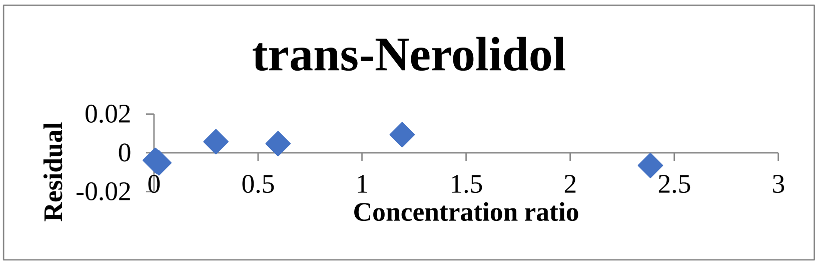
trans-Nerolidol
Residual
0.02
0
-0.02
0
0.5
1
1.5
2
2.5
3
Concentration ratio

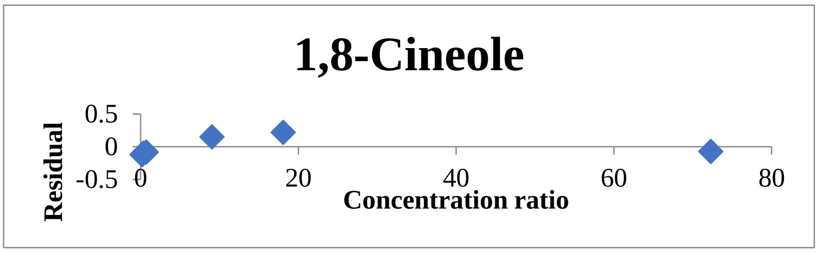
1,8-Cineole
Residual
0.5
0
-0.5
0
20
40
60
80
Concentration ratio

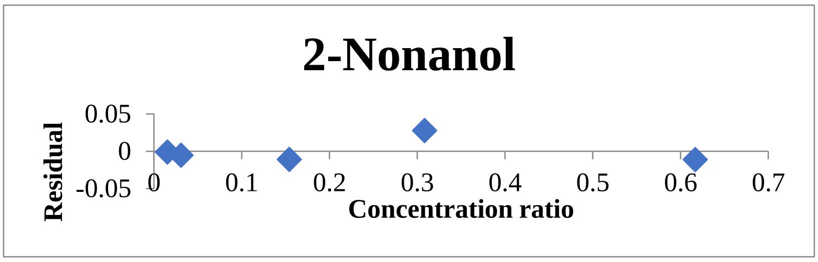
2-Nonanol
Residual
0.05
0
-0.05
0
0.1
0.2
0.3
0.4
0.5
0.6
0.7
Concentration ratio

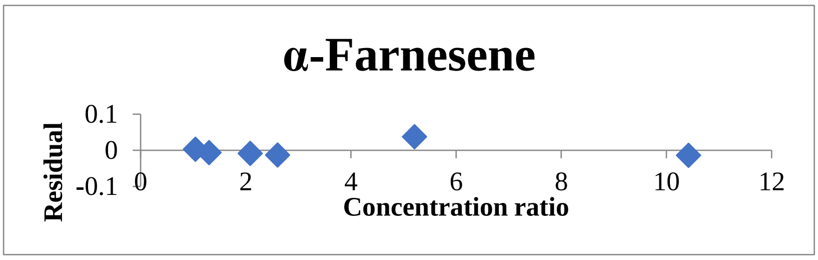
α-Farnesene
Residual
0.1
0
-0.1
0
2
4
6
8
10
12
Concentration ratio

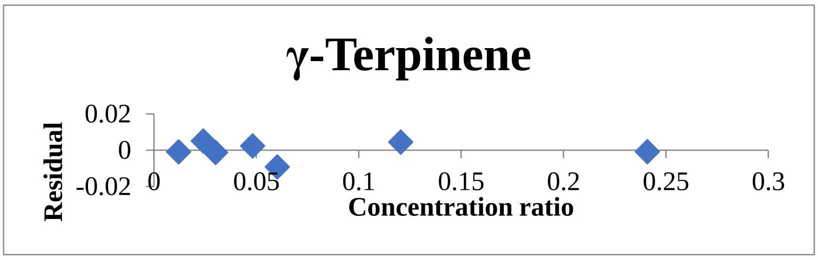
γ-Terpinene
Residual
0.02
0
-0.02
0
0.05
0.1
0.15
0.2
0.25
0.3
Concentration ratio

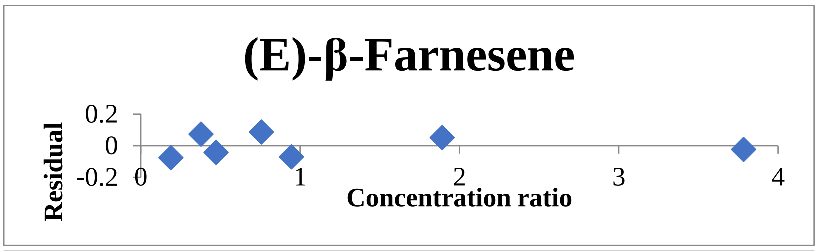
(E)-β-Farnesene
Residual
0.2
0
-0.2
0
1
2
3
4
Concentration ratio

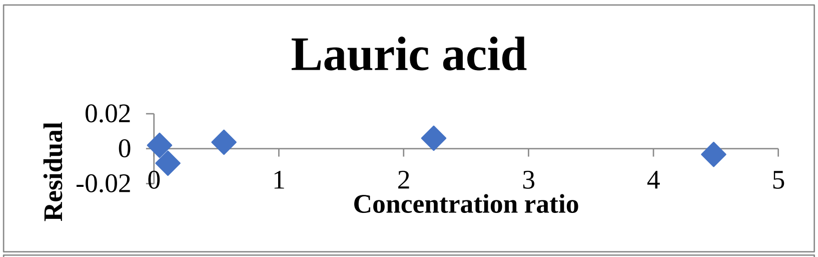
Lauric acid
Residual
0.02
0
-0.02
0
1
2
3
4
5
Concentration ratio

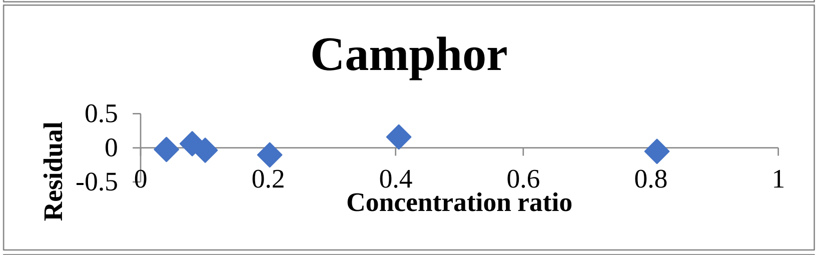
Camphor
Residual
0.5
0
-0.5
0
0.2
0.4
0.6
0.8
1
Concentration ratio

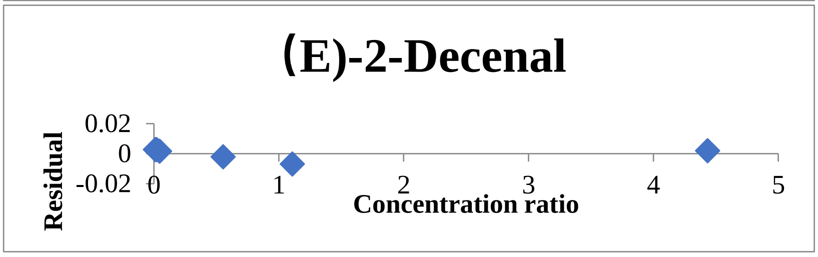
(E)-2-Decenal
Residual
0.02
0
-0.02
0
1
2
3
4
5
Concentration ratio

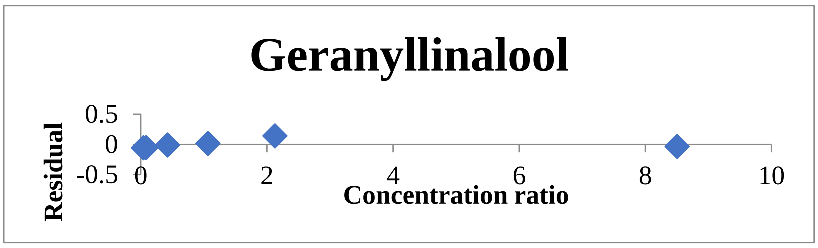
Geranyllinalool
Residual
0.5
0
-0.5
0
2
4
6
8
10
Concentration ratio

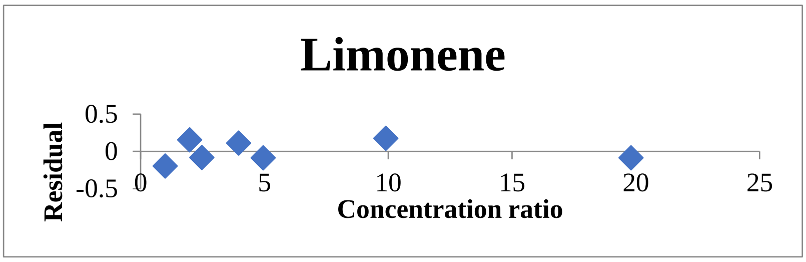
Limonene
Residual
0.5
0
-0.5
0
5
10
15
20
25
Concentration ratio

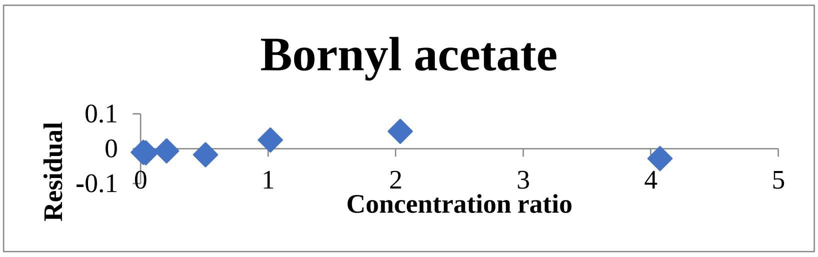
Bornyl acetate
Residual
0.1
0
-0.1
0
1
2
3
4
5
Concentration ratio

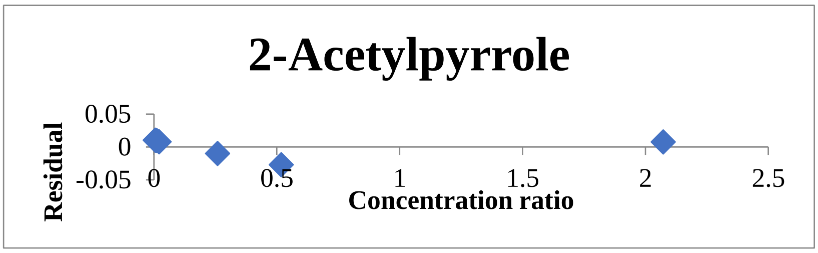
2-Acetylpyrrole
Residual
0.05
0
-0.05
0
0.5
1
1.5
2
2.5
Concentration ratio

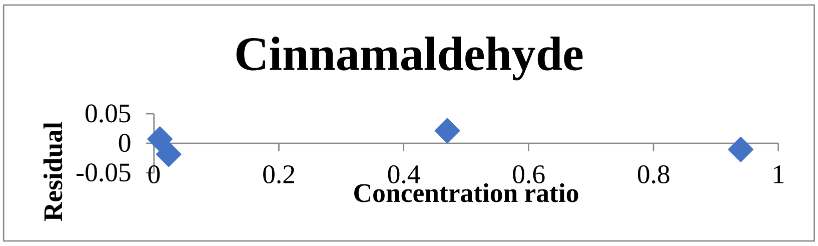
Cinnamaldehyde
Residual
0.05
0
-0.05
0
0.2
0.4
0.6
0.8
1
Concentration ratio

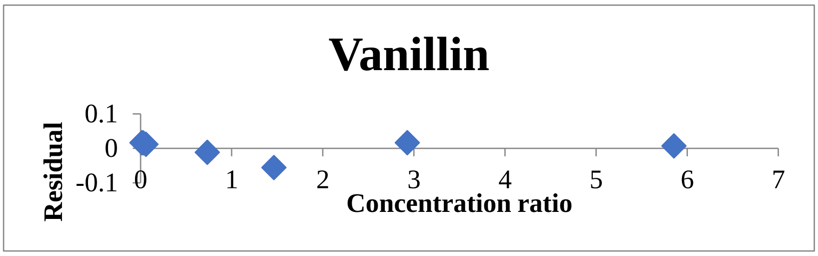
Vanillin
Residual
0.1
0
-0.1
0
1
2
3
4
5
6
7
Concentration ratio

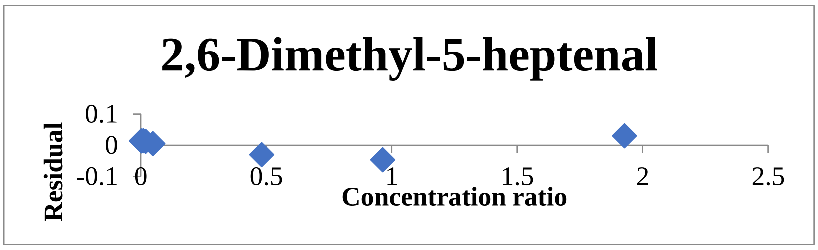
2,6-Dimethyl-5-heptenal
Residual
0.1
0
-0.1
0
0.5
1
1.5
2
2.5
Concentration ratio

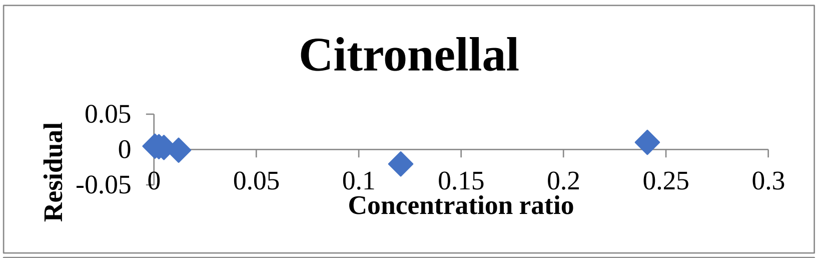
Citronellal
Residual
0.05
0
-0.05
0
0.05
0.1
0.15
0.2
0.25
0.3
Concentration ratio

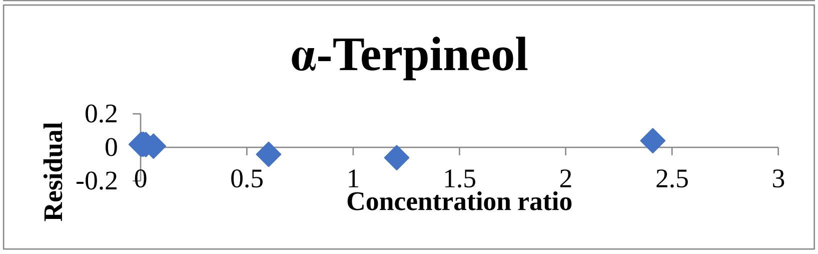
α-Terpineol
Residual
0.2
0
-0.2
0
0.5
1
1.5
2
2.5
3
Concentration ratio

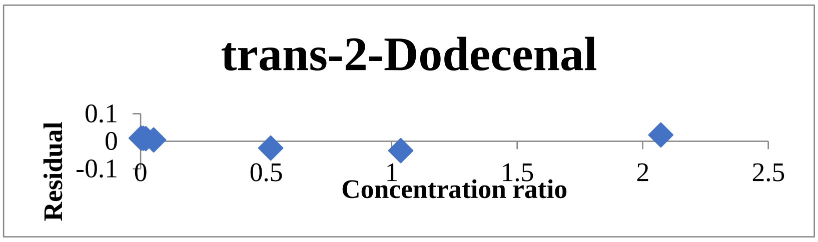
trans-2-Dodecenal
Residual
0.1
0
-0.1
0
0.5
1
1.5
2
2.5
Concentration ratio

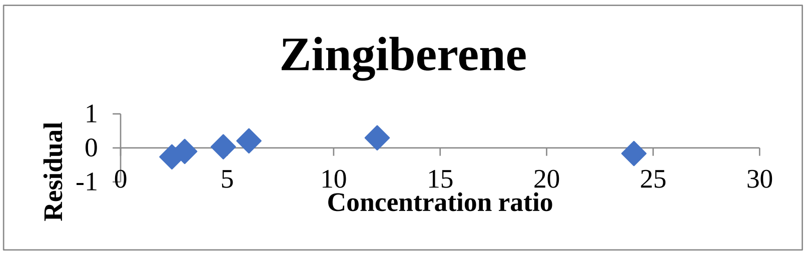
Zingiberene
Residual
1
0
-1
0
5
10
15
20
25
30
Concentration ratio
